# Induction of migration of periodontal ligament cells by selective regulation of integrin subunits

**DOI:** 10.1111/jcmm.14023

**Published:** 2018-12-03

**Authors:** Mari Kawamura, Tadashi Yamamoto, Keisuke Yamashiro, Shinsuke Kochi, Chiaki Yoshihara‐Hirata, Hidetaka Ideguchi, Hiroaki Aoyagi, Kazuhiro Omori, Shogo Takashiba

**Affiliations:** ^1^ Department of Pathophysiology ‐ Periodontal Science Okayama University Graduate School of Medicine, Dentistry and Pharmaceutical Sciences Okayama Japan

**Keywords:** extracellular matrix, integrin, microenvironment, migration, periodontal ligament cells

## Abstract

The recruitment of tissue‐resident stem cells is important for wound regeneration. Periodontal ligament cells (PDL cells) are heterogeneous cell populations with stemness features that migrate into wound sites to regenerate periodontal fibres and neighbouring hard tissues. Cell migration is regulated by the local microenvironment, coordinated by growth factors and the extracellular matrix (ECM). Integrin‐mediated cell adhesion to the ECM provides essential signals for migration. We hypothesized that PDL cell migration could be enhanced by selective expression of integrins. The migration of primary cultured PDL cells was induced by platelet‐derived growth factor‐BB (PDGF‐BB). The effects of blocking specific integrins on migration and ECM adhesion were investigated based on the integrin expression profiles observed during migration. Up‐regulation of integrins α3, α5, and fibronectin was identified at distinct localizations in migrating PDL cells. Treatment with anti‐integrin α5 antibodies inhibited PDL cell migration. Treatment with anti‐integrin α3, α3‐blocking peptide, and α3 siRNA significantly enhanced cell migration, comparable to treatment with PDGF‐BB. Furthermore, integrin α3 inhibition preferentially enhanced adhesion to fibronectin via integrin α5. These findings indicate that PDL cell migration is reciprocally regulated by integrin α3‐mediated inhibition and α5‐mediated promotion. Thus, targeting integrin expression is a possible therapeutic strategy for periodontal regeneration.

## 1 INTRODUCTION

Periodontitis is an oral biofilm‐induced chronic inflammatory disease involving the loss of supporting connective tissue and alveolar bone around the teeth, and it is one of the most prevalent infectious diseases worldwide.^1^ For decades, the ultimate goal of periodontal therapies has been to achieve regeneration of damaged tissue. Wound healing following periodontal therapy consists of three phases: inflammation, granulation tissue formation and matrix remodeling, similar to wound healing of other tissues. However, it is noteworthy that periodontal tissue contains periodontal ligaments (PDL) between two types of calcified tissues—tooth root cementum and alveolar bone—and has dual characteristics of calcification and non‐calcification. Therefore, the regeneration of periodontal tissue requires the orchestration of several different cell types, including PDL cells, cementoblasts, bone cells and gingival epithelial cells. The healing patterns depend on which of these cell types can predominantly migrate into the wound site to reconstruct the periodontal defects. The recruitment of residential tissue stem cells and subsequent proliferation and differentiation is important to achieve the regeneration of the wound tissue.^2^, ^3^

Periodontal ligament is important in periodontal tissue homoeostasis as a mechanical cushion supporting the teeth against occlusal force. PDL serves as a fibrous attachment around the tooth root with vascular and nerve supplies. Moreover, PDL cells contain heterogeneous fibroblast populations and play a critical role in the regeneration of periodontal tissue by providing multi‐potent stem cells and osteogenic progenitor cells capable of regenerating cementum, bone, and the connective tissue itself.^4^, ^5^ To exert these important biological roles, it is crucial to accelerate the migration and adhesion of residential PDL cells on the root surface of intra‐bony defects.

All cell behaviours, including migration, are induced by changes in the local biochemical and mechanical microenvironment that are promoted by the coordinated interactions between growth factors, nearby niche cells and the extracellular matrix (ECM).^6^ Many growth factors have been extensively investigated for their regenerative properties related to the migration of periodontal tissue‐resident cells. However, the effects of these growth factors remain limited and unpredictable. Therefore, a new strategy is expected to improve the biological stability, spatiotemporal specificity and cost‐effectiveness of the agents.^7^, ^8^ Residential PDL cells migrate and proliferate into wounds, and they begin to deposit abundant ECM components during the final phase of wound healing. Progenitor and stem cells are highly sensitive to the intrinsic properties of the ECM.^9^ The ECM acts as a reservoir for soluble growth factors and mediates essential signals for wound healing. Therefore, in addition to growth factors, the modulation of the ECM microenvironment is important for the recruitment of PDL cells to the periodontal defects and induction of their biological effects.^10^

Integrins provide essential intercellular signals for cell migration by supporting adhesion to the ECM. This mechanism involves adaptors that link with the actin cytoskeleton and regulate cell polarity during migration in various cell types.^11^ Moreover, integrin‐mediated signals modulate growth factor‐induced intracellular signalling by regulating the distribution and activation of growth factors.^12^ Integrins are αβ heterodimers. Eight β subunits can interact with 18 α subunits to form 24 distinct integrins, which can be classified into several subfamilies based on their ECM ligand specificity.^13^ PDL cells express multiple integrins (α1β1, α2β1, α3β1, α4β1, α5β1, α11β, αvβ3 and αvβ5). Many of these integrins show altered expression patterns in periodontal disease tissue.^14^ The selective regulation of each integrin subunit may modulate the migration of PDL cells; however, little is known about the respective roles of integrin subunits and the molecular mechanisms governing the ECM microenvironment during PDL cell migration. Furthermore, the specific mechanisms of PDL cell recruitment to wound sites have yet to be elucidated. Recently, pharmacological inhibitors, such as monoclonal antibodies and peptide analogues for integrin subunits, have been used as treatment options in various diseases, including cancer, infection, thrombosis and autoimmune disorders.^15^ Therefore, understanding the mechanisms of adhesion and migration of PDL cells as a function of different integrin subunits is important in developing new strategies for periodontal treatment.

In this study, we examined the spatiotemporal expression profiles of integrins and the ECM during growth factor‐mediated migration of PDL cells and determined the adhesion molecules involved in this process. Moreover, the regulation of specific integrin subunits involved in PDL cell migration was investigated using integrin‐neutralizing antibodies, integrin‐blocking peptides and integrin‐siRNAs.

## 2 MATERIALS AND METHODS

### 2.1 Cell culture and reagents

Periodontal ligament samples were obtained from six donors with healthy periodontal tissue surrounding their extracted third molars or premolars after informed consent was obtained. The study was approved by the Ethics Committee at Okayama University Graduate School of Medicine, Dentistry and Pharmaceutical Sciences and Okayama University Hospital (no. 2070). Fibroblastic PDL cells were obtained by enzymatic digestion and maintained in α‐modified minimum essential medium (α‐MEM; Sigma‐Aldrich, St. Louis, MO, USA) supplemented with 20% foetal bovine serum (FBS; Biowest, Logan, UT, USA), 2 mmol/L L‐glutamine (Thermo Fisher Scientific, Waltham, MA, USA), and 100 U/mL penicillin‐streptomycin antibiotic mixture (Thermo Fisher Scientific) up to their fifth passage as described previously.^16^ PDL cells were starved with 0.1% FBS for 24 hours, seeded at 3.75 × 10^4^ cells/cm^2^, and cultured for 9 hours to ensure cell attachment. The cells were then treated with the proliferation inhibitor, mitomycin C (Nacalai Tesque, Kyoto, Japan), for 1 hour before performing the designed experiments. For osteogenic differentiation, subconfluent PDL cells were maintained in osteogenic medium (the above‐mentioned growth medium supplemented with 50 μmol/L ascorbic acid‐2‐phosphate, 10 mmol/L β‐glycerophosphate, and 100 nmol/L dexamethasone) as described previously.^16^ All results were confirmed with at least three independent experiments, each of which was performed in triplicate.

Based on previous reports, several growth factors were selected as candidate migration factors: transforming growth factor‐β1 (TGF‐β1),^17^ fibroblast growth factor‐2 (FGF‐2),^18^ stromal cell‐derived factor‐1 (SDF‐1)^19^ (all from Miltenyi Biotec, Bergisch Gladbach, Germany), bone morphogenetic protein‐2 (BMP‐2)^20^ (R&D System, Minneapolis, MN, USA), and platelet‐derived growth factor‐BB (PDGF‐BB)^21^ (PeproTech, Rocky Hill, NJ, USA).

### 2.2 Migration assay

Cellular migration assays were performed with the Oris^TM^ Cell Migration Assay Kit (Platypus Technologies, Madison, WI, USA) following the manufacturer’s instructions. Briefly, serum‐starved cells were seeded on non‐coated Oris plates (96‐well) for 9 hours until cell attachment was complete and were treated with mitomycin C for 1 hour. The plates were equipped with silicone stoppers to restrict cell seeding to the outer annular regions of the wells. Subsequently, the stoppers were removed from the plate, and cells were re‐fed with medium containing stimulatory factors and 0.1% FBS. After 38 hours of incubation (48 hours after the beginning of culture), the attached cells were stained with 0.1 μmol/L Corning^TM^ Calcein AM Fluorescent Dye (Thermo Fisher Scientific). The cellular migration area was visualized using a fluorescence microscope (BZ‐X700; KEYENCE, Osaka, Japan) at a magnification of 4× and quantified by Image J software (NIH, Bethesda, MD, USA).

### 2.3 Time‐lapse microscopy

Growth factor‐treated PDL cells on the Oris plate were placed in the temperature‐controlled environment of the Cellomics Array Scan VTI (Thermo Fisher Scientific) for 38 hours, and time‐lapse images of PDL cell migration were recorded in the bright field at 10 frames/s according to the manufacturer’s instructions. Recordings were played back and digitized on a computer.

### 2.4 Real‐time RT‐PCR

Periodontal ligament cells from a single donor were treated with growth factors and harvested after 8 hours. Aliquots of total RNA (1 μg) were recovered from the cells using the RNeasy Mini Kit and gDNA eliminator spin columns (Qiagen, Hilden, Germany) in accordance with the manufacturer’s protocol. PCR analysis of the expression of 84‐spotted cell adhesion genes was performed with the RT^2^ Profiler™ PCR Array Human Extracellular Matrix & Adhesion Molecules (PAHS‐013Z; Qiagen) according to the manufacturer’s protocol. The data were generated by the PCR Array Data Analysis Web Portal (version 3.5) using the default set format. Five endogenous control genes, β‐actin, β2‐microglobulin, glyceraldehyde‐3‐phosphate dehydrogenase (*GAPDH*), hypoxanthine phosphoribosyltransferase 1, and ribosomal protein, large P0, were used for data normalization. For quantitative RT‐PCR analyses, RNA from six donors was reverse transcribed to cDNA by SuperScript^TM^ III (Thermo Fisher Scientific). The PCR analyses were performed by the ^∆∆^Ct method as previously described.^16^ Gene‐specific primers were designed using Primer3 (https://bioinfo.ut.ee/primer3-0.4.0/primer3/) and are described in Table S1. Relative expression was shown after normalization relative to expression of the *GAPDH* mRNA. The amplification conditions consisted of an initial 10 minutes of denaturation at 95°C, followed by 40 cycles of denaturation at 95°C for 10 seconds, annealing at 60°C for 15 seconds and elongation at 72°C for 20 seconds.

### 2.5 Immunoblot analysis

Periodontal ligament cells were treated with growth factors and harvested after 38 hours. Aliquots of total protein (40 μg) from each sample were subjected to immunoblotting as described previously^16^ using antibodies specific to integrin α3 (1:500; Sigma‐Aldrich), integrin α4 (1:1000; Cell Signaling, Beverly, CA, USA), integrin α5 (1:1000; Abcam, Cambridge, MA, USA), pro‐collagen type I (1:1000; Developmental Studies Hybridoma Bank), fibronectin (1:500; Abcam), vitronectin (1:1000; Proteintech Group, Rosemont, IL, USA), and GAPDH (1:3000; Cell Signaling) that served as a loading control. The signal intensities were quantified by densitometric analysis using Image J.

### 2.6 Immunofluorescence staining

Periodontal ligament cells were treated with growth factors, harvested after 38 hours, and fixed in 3.7% formaldehyde in phosphate‐buffered saline (PBS). The samples were subsequently incubated with 1:100 dilution of primary antibodies for Golgi apparatus (MBL, Nagoya, Japan), integrin α3 (Sigma‐Aldrich), integrin α5 (Abcam), fibronectin (Abcam), laminin‐5 (Abcam) and vitronectin (Proteintech Group), followed by the addition of a 1:200 dilution of Alexa Fluor 488‐ or 594‐labelled secondary antibodies (Thermo Fisher Scientific). Negative control samples were incubated with an isotype‐control IgG antibody (Cell Signaling) in place of the primary antibody. Nuclear staining was performed with 4′,6‐diamidino‐2‐phenylindole (DAPI; Vector Laboratories, Burlingame, CA, USA). Staining signals were visualized using a confocal fluorescence microscope (ZEISS LSM780; Carl Zeiss, Oberkochen, Germany). The composite image was obtained by superimposing the images from different fluorescent channels. The *x‐z* axis images (vertical sections) of the cells were acquired by reconstructing the *x‐y* images using the ZEN 2012 software Ver.1.1.2.0 (Carl Zeiss).

### 2.7 Inhibition of integrin function

To block integrin function, neutralizing antibodies for integrin α3 and integrin α5 (both from Sigma‐Aldrich) and isotype‐control antibodies (Cell Signaling) were used. For peptide inhibition, peptides homologous to the β‐propeller repeat regions of the extracellular domains of the integrin α3 chain (AA 273‐289), α325 (PRHRHMGAVFLLSQEAG, one‐letter code for the amino acid) and the scrambled control peptide, Sc α325 (HQLPGAHRGVEARFSML), were used (AnaSpec, Fremont, CA, USA). α325 inhibits integrin α3 signalling by disrupting the interaction between integrin α3 and urokinase receptor (uPAR).^22^ For siRNA inhibition, Silencer^®^ Select siRNA (Thermo Fisher Scientific) was used. Integrin α3 siRNA was designed to target against human integrin α3 mRNA (GenBank NM_002204.2). The oligo sequences were as follows: oligonucleotide 1 (siRNA ID: s7541; sense: 5’‐GUAAAUCACCGGCUACAAAtt‐3’, antisense: 5’‐UUUGUAGCCGGUGAUUUCca‐3’), oligonucleotide 2 (siRNA ID: s7542; sense: 5’‐CAACGUGACUGUGAAGGCAtt‐3’, antisense: 5’‐UGCCUUCACAGUCACGUUGgt‐3’). Silencer^TM^ Select Negative Control No. 1 siRNA (Thermo Fisher Scientific) was used as a non‐targeting control. PDL cells (1 × 10^6^ cells) were cultured in 6‐well dish for 24 hours and transfected with Lipofectamine^TM^ RNAiMAX Transfection Reagent in Opti‐MEM^®^ (both from Thermo Fisher Scientific) according to the manufacturer’s protocol. After 24 hours of transfection, PDL cells were harvested to measure the transfection efficacy by RT‐PCR and subsequent analysis was performed. For migration and adhesion assay, control PDL cells were sham treated with Lipofectamine only.

### 2.8 Cell adhesion assay

Adhesion assays were performed as previously described^23^ to examine the effects of integrin α3 inhibition on PDL cell adhesion. Briefly, 96‐well plates (Corning, New York, NY, USA) were coated with either 10 μg/mL human fibronectin (FN; #F‐4759; Sigma‐Aldrich), human vitronectin (VN; #AF‐140‐09; PeproTech) or bovine serum albumin (BSA; Sigma‐Aldrich) for 12 hours at 4°C. After washing three times with PBS, the plates were blocked with 1% BSA at 25°C for 1 hour. For peptide inhibition, subconfluent PDL cells were trypsinized and resuspended in culture medium with either α325, Sc α325 (10 μg/mL), or the equivalent volume of solvent (sterile water) and incubated for 10 minutes on ice. For siRNA inhibition, transfection using Integrin α3 siRNA (s7541) and Negative Control No. 1 siRNA was performed as described above. Subsequently, the integrin α3‐inhibited PDL cells were seeded in the coated plates at a density of 7.5 × 10^4^ cells/cm^2^. After incubation for 1 hour at 37°C, non‐adherent cells were washed away three times with PBS. The adherent cells were fixed with 3.7% formaldehyde in PBS and stained with DAPI. The stained images were captured by fluorescence microscopy at 4× magnification and an exposure time of 1/30 seconds (BZ‐X700; KEYENCE) using the Oris™ Detection Mask to restrict visualization to 2‐mm diameters in the middle of each well. The number of DAPI‐positive cells was determined using Image J.

### 2.9 Statistical analysis

The data are presented as the mean ± SD from at least three independent experiments. One‐way ANOVA was used to test the difference between three or more groups, and a multiple comparison test was further conducted by the Tukey‐Kramer test. The Student’s *t* test was used to evaluate statistical differences between two groups. Statistical analysis was carried out using the JMP Statistics Software Package (SAS Institute, Cary, NC, USA), and *P* < 0.05 indicates statistical significance.

## 3 RESULTS

### 3.1 Induction of migration in PDL cells

**Figure 1:**
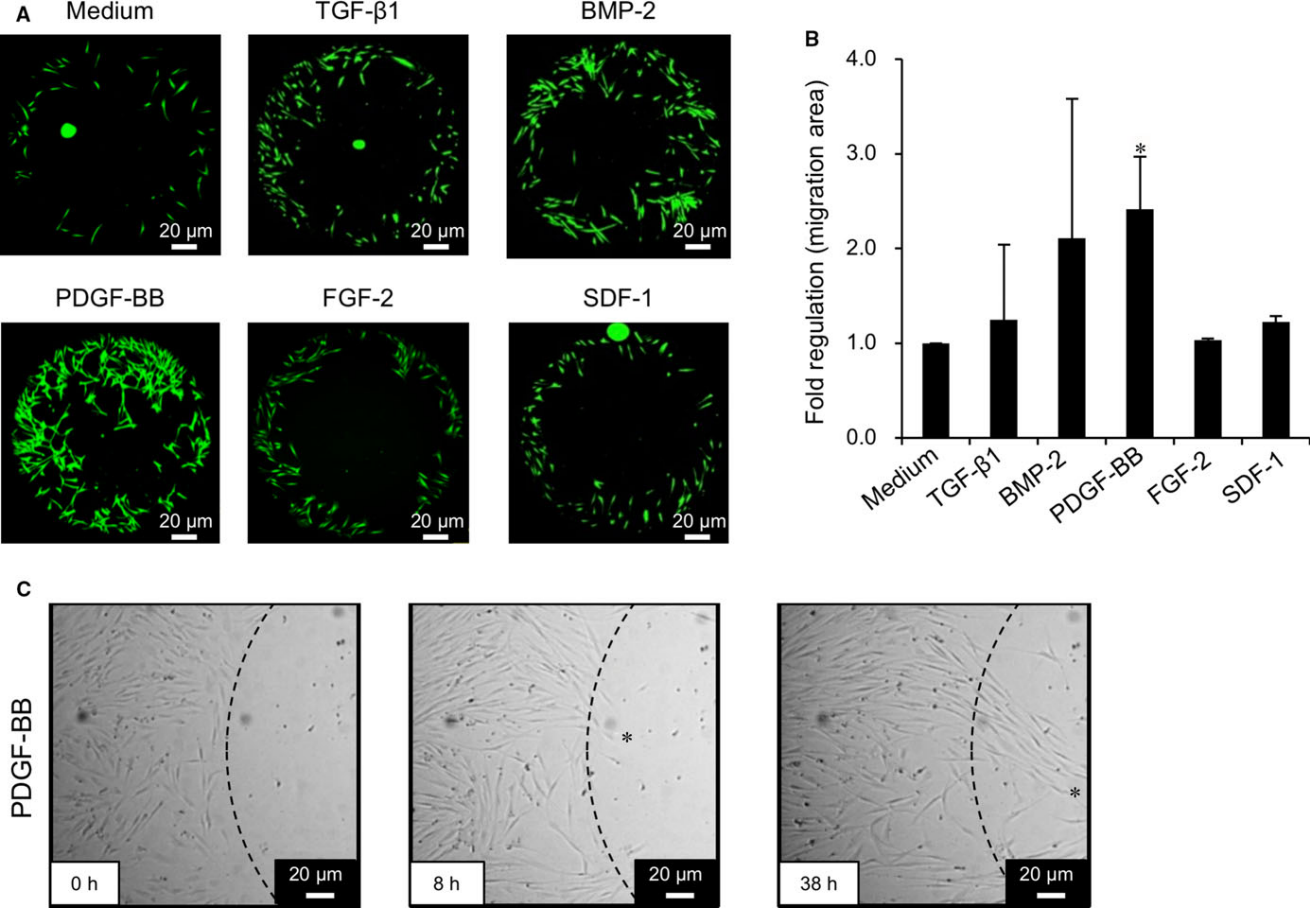
Induction of cellular migration by growth factors. (A) PDL cells were stimulated for 38 h with TGF‐β1 (10 ng/mL), BMP‐2 (100 ng/mL), PDGF‐BB (10 ng/mL), FGF‐2 (10 ng/mL), and SDF‐1 (100 ng/mL) to initiate migration. Representative images of calcein‐stained PDL cells in the designated migration area are indicated. Scale bar: 20 μm. (B) Altered migration areas were quantified using Image J software. The fold increase relative to the migration area of control PDL cells (medium without growth factors) is shown on the *y*‐axis. n = 4 (PDL cells from four donors), *P < 0.05 vs medium, ANOVA/Tukey‐Kramer test. (C) Typical time‐lapse images of PDL cell migration at 0, 8 and 38 h after stimulation with PDGF‐BB (10 ng/mL). Leader cells guiding the collective migration of cells are indicated (*). Scale bar: 20 μm

Several growth factors were selected as candidate migration factors. The optimal concentrations of each growth factor were determined as follows: 10 ng/mL TGF‐β1, 100 ng/mL BMP‐2, 10 ng/mL PDGF‐BB, 10 ng/mL FGF‐2, and 100 ng/mL SDF‐1. Optimal concentrations were based on previous reports and data from the 3‐(4,5‐dimethylthiazol‐2‐yl)‐5‐(3‐carboxymethoxyphenyl)‐2‐(4‐sulfophenyl)‐2H tetrazolium (MTS) assay (Figure S1A). Subsequent migration assays indicated that PDGF‐BB was the most effective among the growth factors examined. The migration area of PDL cells increased significantly in PDGF‐BB‐treated cells, when compared to controls (2.4‐fold, *P < *0.05, Figure 1A, B). Therefore, 10 ng/mL PDGF‐BB was used as a migration‐inducing factor for all experiments in this study.

Time‐lapse analyses indicated that PDL cells began to migrate after 8 hours of PDGF‐BB stimulation following pre‐treatment with 1 μg/mL mitomycin C, which caused no cell toxicity (Figure S1B). The cell movement was gradual until 24 hours; thereafter, diffuse migration of leader cells guiding the collective migration of cells^24^ was observed after 38 hours (Figure 1C).

### 3.2 Gene expression profiles of migrating PDL cells

**Figure 2:**
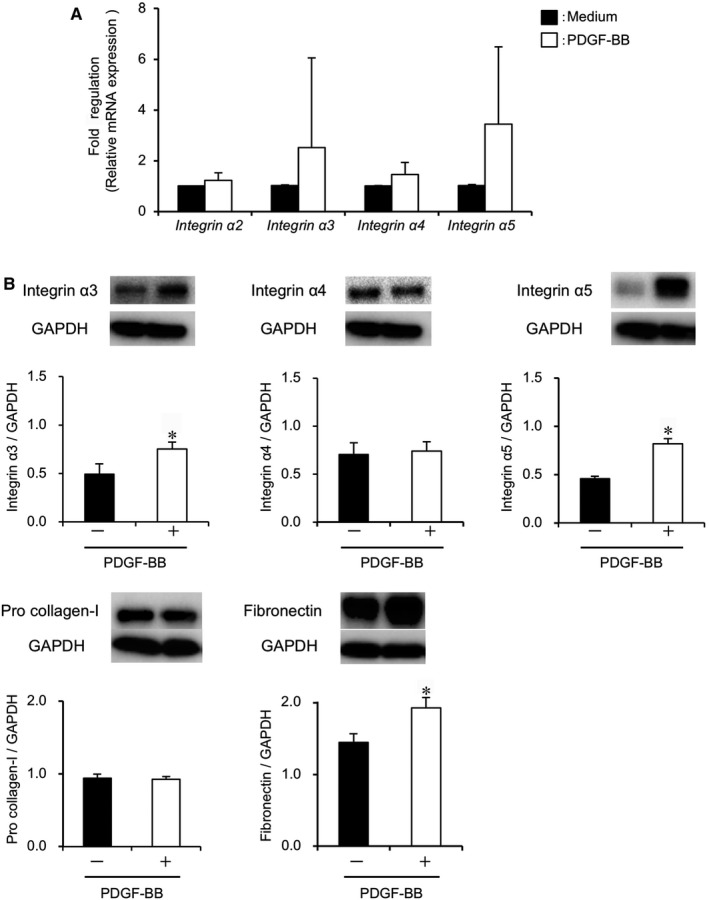
Differential expression of integrins and ECM during PDGF‐BB‐mediated migration. PDL cells were stimulated with 10 ng/mL PDGF‐BB (+; open bars) or medium‐only (−; solid bars) and harvested after 8 h for mRNA analysis and after 38 h for protein analysis. (A) Real‐time RT‐PCR analysis: Quantities of integrin α2, α3, α4 and α5 mRNA were determined relative to *GAPDH* by the ^∆∆^Ct method and are shown as fold induction on the *y*‐axis. *Integrin *α*3* and α*5* tended to increase although there was no statistical significance. n = 6 (PDL cells from six donors). (B) Immunoblot analysis: The protein levels of integrin α3, α4, α5, pro‐collagen type I and fibronectin were normalized to GAPDH levels by densitometric analysis, and the relative expression is shown on the *y*‐axis. n = 3 (PDL cells from three donors), *P < 0.05 vs medium, Student’s *t* test

We performed PCR array analysis for the initial migration of PDL cells with vs without PDGF‐BB treatment after 8 hours (from Figure 1C). The mRNA accumulation levels were relatively high (threshold cycle (Ct) <30) among the 84 selected genes (Figure S2A). The data indicated that 15 genes were differentially expressed greater than twofold in PDL cells treated with PDGF‐BB as compared with control cells. Of these, genes encoding four subunits of integrin, α2 (*ITGA2*), α3 (*ITGA3*), α4 (*ITGA4*) and α5 (*ITGA5*), were elevated by approximately twofold. However, the expression of their binding β subunit, integrin β1 (*ITGB1*), did not change (Table 1). The genes encoding major ECM‐ligands of the integrins,^25^ namely collagen type I (*COL1A1*) for integrin α2, laminin 5 (*LAMA3*, *LAMB3*) for integrin α3, vascular cell adhesion molecule 1 (*VCAM1*) for integrin α4 and fibronectin (*FN1*) for integrin α5, were also examined. Although high expression of *FN1* and *COL1A1* was observed (Ct <17), the transcript levels were similar between PDL cells treated with PDGF‐BB and controls. The expressions of *LAMA3*, *LAMB3* and *VCAM1* were relatively low (Ct = 23‐29). *LAMB3* expression increased by approximately twofold, whereas *LAMA3* decreased greater than twofold. Integrin expression in PDL cells from different six donors was verified by RT‐PCR (Figure 2A). Although there was no significant difference because of variations in the levels of each integrin among the donors, the expression of *ITGA3* and *ITGA5 *tended to be higher in PDL cells treated with PDGF‐BB when compared to control cells.

**Table 1:** Differentially expressed genes regulated by PDGF‐BB in PDL cells

Gene symbol	Ct		Fold regulation
	Medium	PDGF‐BB	
*FN 1*	16.1	14.3	−1.32
*Col I A1*	16.5	14.7	−1.31
*ITGB1*	18.6	16.1	1.18
*ITGA5*	22.2	19.0	1.91
*ITGA3*	24.1	20.5	2.56
*ITGA2*	26.0	22.5	2.45
*ITGA4*	26.0	22.8	2.00
*LAMB3*	26.4	23.4	1.73
*LAMA3*	28.4	27.3	−2.22
*VCAM 1*	29.8	28.1	−1.41

PCR array analysis of the expression of 84‐spotted cell adhesion genes in PDL cells with or without PDGF‐BB. The threshold cycle (Ct) and the fold changes in gene expression with PDGF‐BB (+, up‐regulation; –, down‐regulation) are presented. Genes encoding four subunits of integrins, α2 (*ITGA2*), α3 (*ITGA3*), α4 (*ITGA4*) and α5 (*ITGA5*), were elevated by approximately twofold. Their specific ECM‐ligands, fibronectin 1 (*FN1*), collagen type I alpha 1 (*COL1A1*), laminin subunit beta 3 (*LAMB3*), laminin subunit alpha 3 (*LAMA3*), vascular cell adhesion molecule 1 (*VCAM1*), are also listed.

Protein quantification by immunoblotting indicated that the relative expression levels (ratio to GAPDH) of integrin α3 (medium: 0.5 ± 0.1, PDGF‐BB: 0.8 ± 0.1, *P < *0.05) and integrin α5 (medium: 0.5 ± 0.02, PDGF‐BB: 0.9 ± 0.1, *P < *0.05) were increased in PDL cells treated with PDGF‐BB compared with control cells (Figure 2B). The ECM ligand, fibronectin, was also significantly increased in PDGF‐BB‐treated PDL cells (medium: 1.4 ± 0.1, PDGF‐BB: 1.9 ± 0.1, *P < *0.05). However, integrin α4 and type I pro‐collagen remained at similar levels. Laminin‐5 was not detected in either group of PDL cells (Figure S2B).

### 3.3 Subcellular localization of integrin subunits and ECM in migrating PDL cells

**Figure 3:**
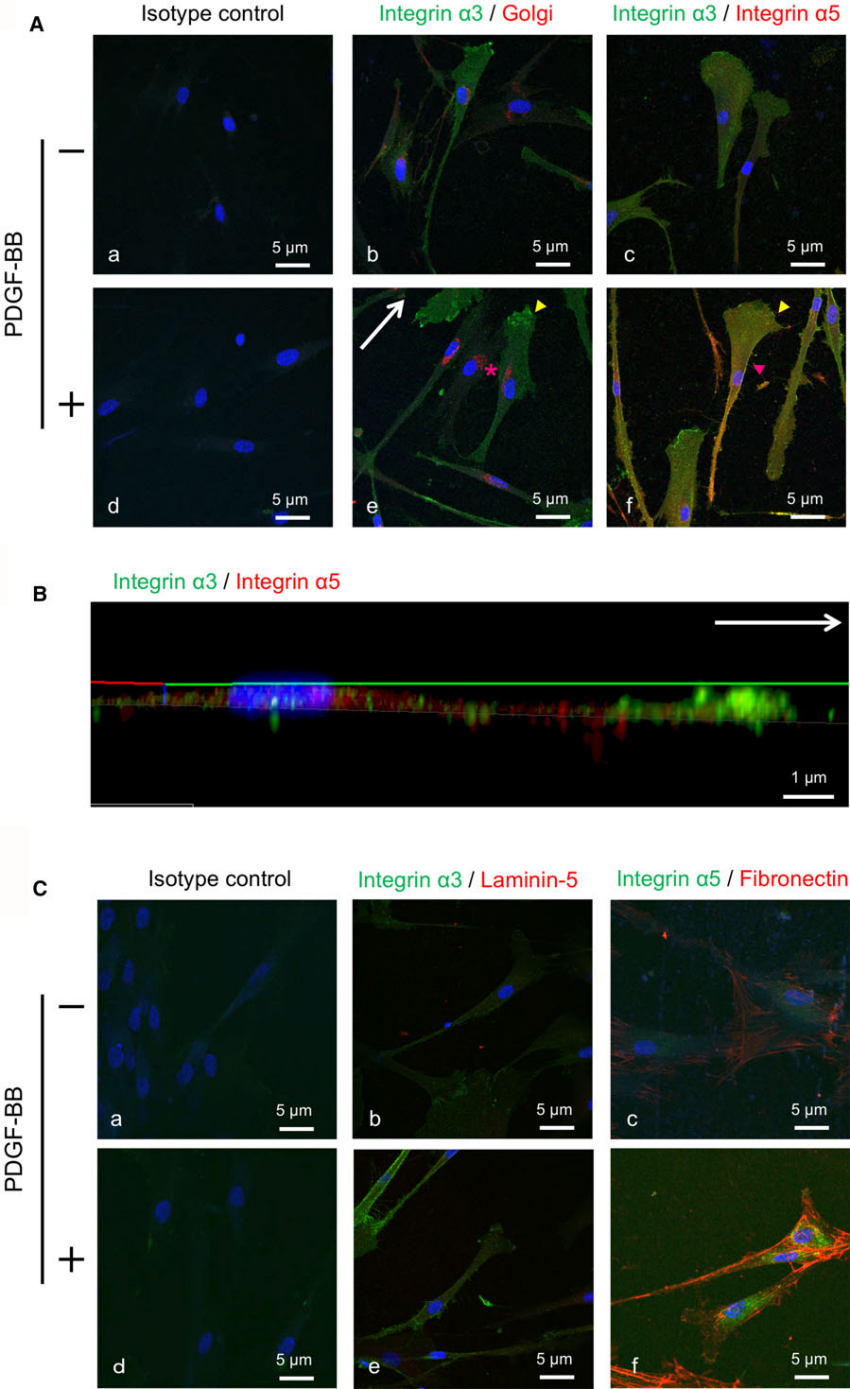
Subcellular localization of integrins and ECM during PDGF‐BB‐mediated migration. PDL cells were stimulated with 10 ng/mL PDGF‐BB (+) or medium‐only (−) for 38 h, and immunofluorescence analysis was performed. Overlays of the images are shown with co‐localization depicted in yellow. Nuclei are stained with DAPI (blue). PDL cells from three donors were used, one for 3D confocal microscopy. Representative images are shown. (A) The localization of Golgi apparatus (red) and integrin α3 (green) are shown in b and e. Staining of the Golgi apparatus (red asterisk) is observed in front of the nucleus in the direction of migration (white arrow). Integrin α3 staining is observed prominently at the leading edge (yellow arrowhead). The differential localizations of integrin α5 (red) and integrin α3 (green) are shown in c and f. Integrin α5 staining is observed inside of the leading edge (red arrowhead). Negative controls were incubated with isotype control IgG antibody (a, d). Scale bar: 5 μm. (B) Images of *x‐z* planes were obtained by reconstructing the middle region images of *x‐y* planes (direction of migration; white arrow) in migrating PDL cells stimulated with PDGF‐BB. Scale bar: 1  μm. (C) The localizations of laminin‐5 (red) and integrin α3 (green) are shown in b and e. Laminin‐5 immunoreactivity is not detected in integrin α3‐expressing PDL cells with or without PDGF‐BB. The localizations of fibronectin (red) and integrin α5 (green) are shown in c and f. Distinct fibronectin staining is observed in the extracellular region of integrin α5‐expressing PDL cells stimulated with PDGF‐BB. Negative controls were incubated with isotype control IgG antibody (a, d). Scale bar: 5 μm

Cell polarity during migration is represented by localization of the Golgi apparatus.^26^ Immunofluorescence analysis using an anti‐Golgi antibody demonstrated localization of the organelle towards the front of the nucleus in the direction of migration (Figure 3A). Integrin α3 was localized in the cytoplasm and prominently at the leading edge in the membrane protrusions of migrating cells. Integrin α3 staining was higher in PDL cells treated with PDGF‐BB compared with controls. In contrast, integrin α5 staining in PDGF‐BB‐treated cells was increased on the inner side of the leading edge, likely at the base of the lamellipodia, while integrin α5 in control cells was weakly localized in the perinuclear region. Integrin α5 was frequently found associated with fibrous structures behind migrating PDL cells. Confocal immunofluorescence 3D reconstruction of PDL cells confirmed different subcellular localizations of integrin α3 and α5 in migrating PDL cells as demonstrated by the *z*‐plane analysis (Figure 3B). The ECM ligands for integrin α3 and α5, laminin‐5 and fibronectin, respectively, were also examined (Figure 3C). Immunofluorescence analysis indicated no detectable staining of laminin‐5 in the cytoplasm of integrin α3‐expressing PDL cells with or without PDGF‐BB treatment. Increased fibronectin staining was observed in the outer boundary of the plasma membrane of integrin α5‐expressing PDL cells treated with PDGF‐BB as compared with controls.

### 3.4 Regulation of PDL cell migration by integrin‐neutralizing antibodies, blocking peptides, and siRNAs

**Figure 4:**
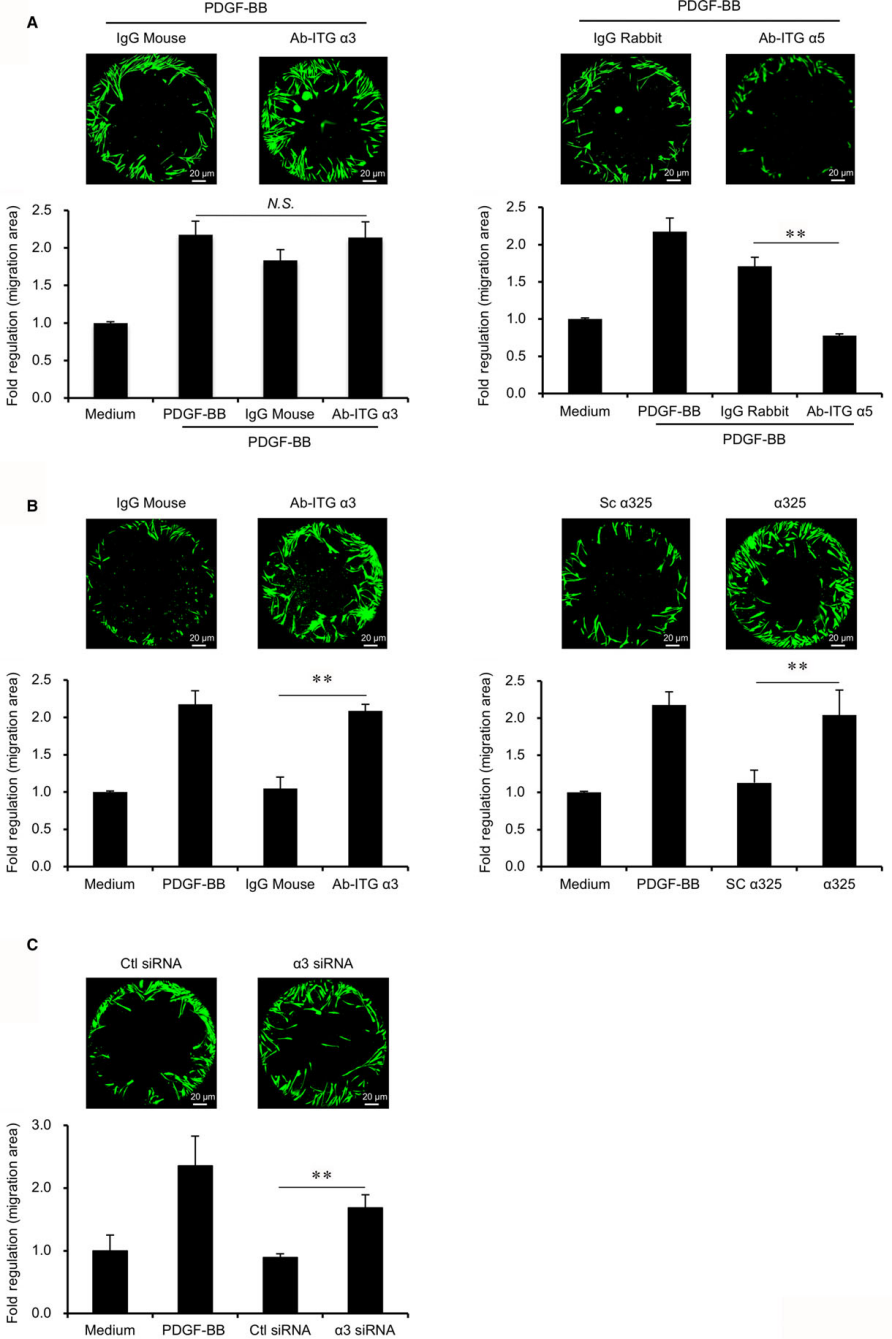
Effects of integrin‐neutralizing antibodies, blocking peptide and siRNA on migration. Migration assays were performed for 38 h using PDL cells stimulated with either integrin‐neutralizing antibodies, isotype‐control antibodies, α325, Sc α325, integrin α3 siRNA, Negative Control siRNA, PDGF‐BB (10 ng/mL) or medium‐only. The fold increase relative to the PDL cell migration area with medium‐only is shown on the *y*‐axis. n = 3 (PDL cells from three donors), **P < 0.001 vs control groups (isotype‐control, Sc α325, or Negative Control siRNA), ANOVA/Tukey‐Kramer test. Representative images of calcein‐stained PDL cells in the designated migration area are indicated at the top of each figure (left panel, control group; right panel, experimental group). Scale bar: 20 μm. (A) PDL cells were stimulated by either neutralizing antibodies (10 μg/mL) for integrin α3 (Ab‐ITG α3), integrin α5 (Ab‐ITG α5), or isotype‐control antibodies (10 μg/mL) with PDGF‐BB. (B) PDL cells were stimulated by either Ab‐ITG α3 (10 μg/mL), the isotype‐control antibody (10 μg/mL), α325 (10 μg/mL) or Sc α325 (10 μg/mL) without PDGF‐BB. (C) PDL cells were transfected by either integrin α3 siRNA (10 nmol/L) or Negative Control siRNA (10 nmol/L) for 24 h and assayed for migration without PDGF‐BB

To inhibit the effects of integrins on the PDGF‐BB‐mediated migration of PDL cells, integrin‐neutralizing antibodies, blocking peptides and siRNAs were used in the migration assay. Optimal concentrations of the inhibitors were determined by the MTS and migration assay (Figure S3). The migration assay indicated that neutralizing antibodies for integrin α5 (Ab‐ITG α5, 10 μg/mL) significantly inhibited PDL cells migration induced by PDGF‐BB (Ab‐ITG α5: 0.8‐fold; isotype‐control: 1.7‐fold, *P < *0.001). In contrast, neutralizing antibodies for integrin α3 (Ab‐ITG α3, 10 μg/mL) tended to increase the migration of PDL cells compared to the isotype‐control, although there was no statistical significance (Figure 4A).

To directly determine the effect of integrin α3 on the behaviour of PDL cells, an inhibition assay was performed without PDGF‐BB‐treatment (Figure 4B). Interestingly, Ab‐ITG α3 significantly enhanced cell migration compared to control untreated PDL cells (Ab‐ITG α3: 2.1‐fold; isotype‐control: 1.0‐fold, *P < *0.001). The migration‐stimulating effect was confirmed using an integrin α3 blocking peptide (α325).^22^ Without PDGF‐BB stimulation, treatment of PDL cells with α325 (10 μg/mL) significantly enhanced cell migration (α325: 2.0‐fold; scrambled peptide [Sc α325]: 1.1‐fold, *P < *0.001). Moreover, the effects of integrin α3 knockdown were examined by integrin α3 siRNA. The efficiency of knockdown was confirmed using two selected siRNA (Figure S4A). The migration of integrin α3‐knockdown PDL cells by 10 nmol/L siRNA (s7141) was enhanced significantly compared to the negative control (α3 siRNA: 1.7‐fold; control siRNA: 0.8‐fold, *P < *0.001) (Figure 4C). Notably, the enhanced levels of migration induced by Ab‐ITG α3, α325, and integrin α3 siRNA were comparable to that induced by treatment with PDGF‐BB. The expression of proliferating cell nuclear antigen showed no change in integrin α3‐knockdown when compared to control PDL cells (Figure S4B). Moreover, Ab‐ITG α3, Ab‐ITG α5 and α325 did not affect MTS activity (Figure S3A, B), indicating that integrin α3 and α5 were not involved in PDL cell proliferation in the culture condition.

### 3.5 Effects of integrin α3 inhibition on osteogenic differentiation of PDL cells

**Figure 5:**
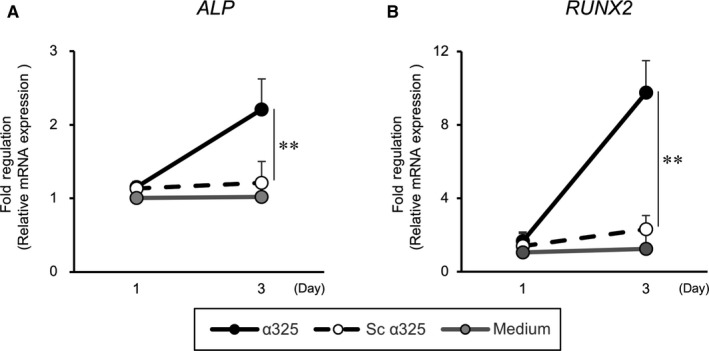
Effects of integrin inhibition on osteogenic gene mRNA expression. After α325 (closed circles) or Sc α325 (open circles) treatment, PDL cells were cultured in osteogenic medium for 1 and 3 days. Quantitative RT‐PCR analyses were performed with the transcribed cDNAs. The mRNA ratio relative to *GAPDH* was calculated (A): *ALP*, (B): *RUNX2.* The fold increase relative to control (medium only: grey circles) is shown on the *y*‐axis, while the *x*‐axis shows the culture period. n = 3 (PDL cells from three donors), *P < 0.001 vs Sc α325, ANOVA/Tukey‐Kramer test

The migration of PDL cells into the wound and subsequent differentiation is important to achieve periodontal regeneration. Therefore, we investigated the effects of integrin α3 inhibition on osteogenic differentiation by analyzing the expression of early osteogenic markers, alkaline phosphatase (*ALP*) and runt‐related transcription factor 2 (*RUNX2*). Treatment of PDL cells with α325 significantly enhanced the gene expression of *ALP* and *RUNX2* at day 3 during osteogenic differentiation (*ALP*: 1.8‐fold; *RUNX2*: 4.2‐fold, *P < *0.001 vs Sc α325) (Figure 5).

### 3.6 Effects of integrin α3 inhibition on PDL cell adhesion to the ECM

**Figure 6:**
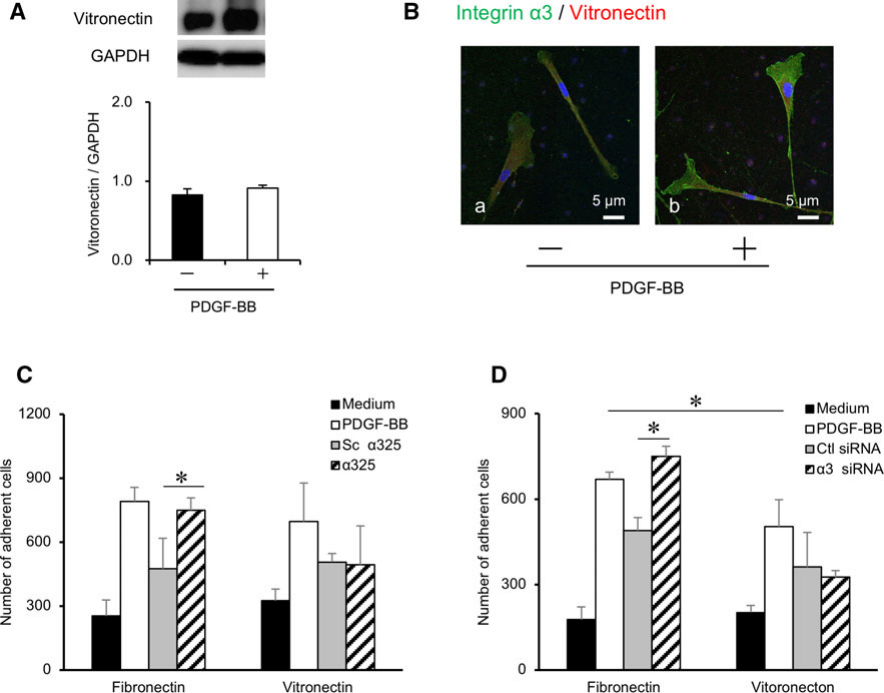
Effects of integrin α3 inhibition on adhesion to fibronectin and vitronectin. (A) The immunoblots for vitronectin are indicated. PDL cells were stimulated with 10 ng/mL PDGF‐BB (+) or medium‐only (−) and harvested after 38 h. n = 3 (PDL cells from three donors). (A) PDL cells were stimulated with 10 ng/mL PDGF‐BB (b) or medium‐only (a) for 38 h, and immunofluorescence analysis was performed. The localizations of integrin α3 (green) and vitronectin (red) are shown. Overlays of the images are shown with co‐localization depicted in yellow. Nuclei are stained with DAPI (blue). PDL cells from three donors were used, and representative images are shown. Scale bar: 5 μm. (C, D) Adhesion assays were performed after 60 min of re‐seeding on the coating plates (fibronectin, vitronectin). The *y*‐axis indicates the number of adherent cells. n = 3 (PDL cells from three donors), *P < 0.05, ANOVA/Tukey‐Kramer test. (C) Resuspended PDL cells were stimulated with 10 μg/mL α325 (lined bars), Sc α325 (grey bars), medium‐only (solid bars) or 10 ng/mL PDGF‐BB (open bars) for 10 min. Stimulation with PDGF‐BB served as a positive control. (D) PDL cells were transfected by either integrin α3 siRNA (10 nmol/L) (lined bars) or Negative Control siRNA (10 nmol/L) (grey bars) for 24 h and assayed for the adhesion assay

Finally, we investigated the interaction of integrin α3 with the ECM‐microenvironment and the molecular mechanisms of α325 or integrin α3 siRNA‐mediated migration of PDL cells. Since no expression of laminin‐5 was detected in PDL cells, we focused on the expression of vitronectin. α325 disrupts the interaction between integrin α3 and uPAR, indirectly inhibiting integrin α3 binding to vitronectin.^22^ The expression of vitronectin was detected in the cytoplasm of integrin α3‐expressing PDL cells by immunoblotting and immunofluorescence analysis. There was no significant difference in the expression between PDGF‐BB‐treated PDL cells and controls (Figure 6A,B). Adhesion assays were performed with α325 or integrin α3 siRNA‐treated PDL cells on FN or VN‐coated plates (Figure 6C,D). PDGF‐BB enhanced adhesion to FN more than to VN (*P < *0.05) (Figure 6D). Interestingly, the adhesion of PDL cells to FN increased remarkably in the presence of α325 or integrin α3 siRNA as compared to controls (vs Sc α325 or Ctl siRNA, *P < *0.05). Furthermore, the stimulatory effect of α325 and integrin α3 siRNA on PDL cell adhesion was comparable to the effect of PDGF‐BB treatment. These data indicated that the enhanced migration of PDL cells by integrin α3 inhibition was predominantly mediated by adhesion to FN. Integrin α3 inhibition tended to decrease the adhesion of PDL cells to VN, although there was no statistical significance.

## 4 DISCUSSION

In this study, our analyses showed the up‐regulation of integrin α3 and α5 in migrating PDL cells treated by PDGF‐BB. The expression profiles and regulatory functions of integrins are quite diverse. Integrin α5 is generally involved in the directional migration of various cell types.^27^ However, integrin α5‐null embryonic cells are still able to migrate on fibronectin‐coated surfaces,^28^ suggesting that integrin α5 is not vital for migration and that its loss is compensated by another integrin subunit. Therefore, it is important to investigate the mechanisms of the adhesion and migration of PDL cells as a function of differential integrin engagement. In PDL cells, there have only been a few studies to examine the mechanisms of integrin‐induced migration. In contrast to our data, increased expression of integrin α5 was shown to be involved in the migration inhibition under inflammatory conditions mediated by tumour necrosis factor‐α.^29^ Glial cell‐derived neurotrophic factor enhances the PDL cell migration via integrin αvβ3.^30^ However, in PCR array analysis, the mRNA levels of integrin αv were similar between PDL cells stimulated with PDGF‐BB and controls. Presumably, the expression patterns and functions of integrins are dependent on the cell type and ECM‐microenvironment during migration. The integrin expression is dynamic and quickly changes even in a slightly different microenvironment.^31^ These may be reasons why our RT‐PCR data indicated variations in the integrin expressions among the donors. Alterations in the expression of individual integrins intricately affect adhesion and migration of PDL cells.

Subpopulations of various integrins in different activation states are localized to the cytoplasm or plasma membrane, depending on factors in the microenvironment, such as the availability of ECM ligands and signalling activators.^11^ Activated integrins preferentially localize to the leading edge, where new adhesions form.^26^ In this study, immunofluorescence analysis indicated that enhanced expression of integrin α3 and α5 were differentially localized in front of migrating PDL cells treated with PDGF‐BB. The localization of integrin α5 changed from a perinuclear region to the base of the lamellipodium after PDGF‐BB stimulation. The localization of α3 remained at the leading edge, although the intensity increased after treatment with PDGF‐BB. In addition, fibronectin expression increased significantly in migrating PDL cells treated with PDGF‐BB, while expression of laminin‐5 was not detected. These data suggest that there are distinct roles for integrin α3 and α5 during PDL cell migration.

We investigated the distinct functions of integrin α3 and α5 during migration. Previous studies indicated that integrin α5‐neutralizing antibodies inhibited the attachment of PDL cells to a cementum attachment protein.^32^ It has also been demonstrated previously that fibronectin induces the migration of PDL cells.^33^ Fibronectin contains an RGD (arginine‐glycine‐aspartic acid) sequence, that is, recognized by integrin α5. Synthetic RGD‐containing peptides promote the adhesion and proliferation of PDL cells.^34^ These studies have indicated that integrin α5 has a crucial role in the fibronectin‐mediated behaviour of PDL cells. Our data showed that integrin α5 was strongly associated with abundant fibronectin fibrils surrounding the migrating PDL cells after treatment with PDGF‐BB. Moreover, integrin α5 antibodies significantly inhibited PDL cell migration. Collagen type I is the major component of periodontal ECM,^4^ and it was detected abundantly in this study. Fibronectin interacts simultaneously with various types of collagens and regulates cell migration by establishing the ECM‐microenvironment.^35^ These data suggest that integrin α5 predominantly promotes directional migration on fibronectin.

Integrin α3 and the binding β subunit, integrin β1, are elements of a laminin receptor with diverse functions. Integrin α3β1 mediates migration of neuronal and tumour cells, while either mediating or inhibiting migration and wound re‐epithelialization in keratinocytes.^36^, ^37^ The role of integrin α3β1 in migration and wound healing is complex and has not yet been fully elucidated. In PDL cells, mRNA and protein expression of integrin α3 have been previously reported^38^; however, the functional role remains unclear. Integrin α3β1 is a receptor for laminin‐5 (α3β3γ2) and α5‐containing laminins, such as laminin‐10 (α5β1γ1) and ‐11 (α5β2γ1).^36^ PCR analysis revealed that the expression of laminin subunit γ1 was significantly low (Ct >30), and laminin subunits α3 and β3 were detected (Ct = 23‐29). However, the laminin‐5 protein was not detected. It remains unclear whether the observed transcriptional changes resulted in parallel changes in protein expression. In the periodontium, laminin localization is limited to the basement membranes of vessels and the epithelium.^39^ The laminin protein is induced only by direct interaction between the epithelial crests of Malassez and PDL cells.^40^

Therefore, we explored another functional ligand of integrin α3 in PDL cells. The cluster of uPAR/vitronectin interacts with integrin α3β1 via β‐propeller, which is distinct from the laminin binding region. uPAR is a cell‐surface receptor for the urokinase‐type plasminogen activator (uPA),^22^ which is constitutively expressed in PDL cells.^41^ uPAR expression is elevated during inflammation, tumour invasion, and tissue remodeling, and it is an important mediator of ECM proteolysis and migration. The functional cluster of integrin α3β1/vitronectin/uPAR is crucial for these processes. α325 inhibits integrin α3β1 signalling by disrupting the cluster and cellular adhesion to vitronectin.^22^, ^42^ In this study, Ab‐ITG α3, α325, and α3 siRNA significantly enhanced PDL cell migration without PDGF‐BB treatment. This migration effect was comparable to the effect of PDGF‐BB treatment. These findings are especially noteworthy since a number of previous studies have reported that α325 suppressed the migration of various cells of epithelial and mesenchymal origin by inhibiting integrin α3β1/uPAR functions.^43^, ^44^

Periodontal ligament cells have several characteristics of calcification, such as the expression of osteogenic genes and the capacity to form mineralized nodules during differentiation.^16^ Treatment of PDL cells with α325 in osteogenic medium demonstrated increased expression of *ALP* and *RUNX2*. Although the underlying mechanism remains unclear, α325 may be feasible candidate peptide to induce migration of PDL cells with osteogenic differentiated status, which is more desirable for efficient hard tissue regeneration. Moreover, α325 was found to have no significant effect on the migration of Ca9‐22 cells derived from gingiva (Figure S5). Owing to the higher proliferative rate of gingival epithelial cells compared to PDL cells, it is ideal that α325 has no stimulatory effect on epithelial migration, thus, allowing tissue defects to be repopulated by PDL cells.^3^

Finally, we investigated the underlying mechanisms behind the unique effects of integrin α3 inhibition on PDL cell migration. Cell adhesion assays indicated that the adhesion of PDL cells to FN was increased by treatment with α325 or α3 siRNA. This effect was comparable to the effect of PDGF‐BB treatment; however, the adhesion to VN showed no significant change or a slight decrease in the presence of α325 or α3 siRNA. These data indicate that the ECM‐mediated migration of PDL cells is reciprocally regulated by specific integrin subunits. Migration is positively regulated by adhesion to FN via integrin α5β1 and negatively regulated by adhesion to VN via integrin α3β1. In the presence of PDGF‐BB, although the expressions of both integrin α3 and α5 were enhanced, the fibronectin‐integrin α5 axis plays a major role in the directional migration of PDL cells.

The molecular interactions of uPAR have been examined previously. Although uPAR consists of corresponding binding sites for α3β1 and α5β1 integrins, it preferentially interacts with α3β1 and promotes cellular adhesion to VN.^22^, ^42^ Moreover, the amount of FN, as well as FN binding, and cell migration induced by α5β1 integrin are also enhanced by uPAR.^45^, ^46^ Therefore, the potential molecular mechanism of PDL cell migration stimulated by α325 involves the selective activation of integrin signalling by uPAR. α325 appears to change the signalling preference of uPAR from integrin α3 to integrin α5. α325 blocks VN adhesion via integrin α3β1 and promotes migration of PDL cells on FN via integrin α5β1. Further investigation is needed to elucidate the exact molecular mechanisms of PDL cell migration by selective regulation of the integrin subunit/uPAR and to define how these mechanisms modulate the ECM microenvironment. As antibody‐based drugs that target integrins have been associated with potentially fatal side‐effect thought to be related to their immunosuppressive properties,^15^ the application of peptide inhibitors that mimic the integrin binding domain could be a promising strategy for therapeutic intervention. Further in vivo assay would be essential to investigate the effects of α325 for periodontal regeneration.

In summary, the results of this study suggest that integrins α3 and α5 play a central role in defining the ECM microenvironment of PDL cells for migration. Our results demonstrated that the expression of integrin α3 and α5 were enhanced in different subcellular localizations and had distinct roles during PDGF‐BB‐mediated migration. Reciprocal functions of these integrins were identified, that is, integrin α5 was stimulatory and integrin α3 was inhibitory for PDL cell migration. Notably, α325, an integrin α3 blocking peptide, was effective for the induction of migration in PDL cells. α325 preferentially blocked VN adhesion via integrin α3β1 and promoted FN adhesion via integrin α5β1. An understanding of the selective regulation of integrin subunits is valuable to provide a detailed molecular picture of the mechanisms of cellular migration. Furthermore, modulation of the integrins expression would be therapeutic strategy, possibly in combination with growth factors, for periodontal tissue engineering and regeneration.

## CONFLICT OF INTEREST

All authors have no conflict of interest regarding this paper.
