# Supplementary figures and images for: Induction of migration of periodontal ligament cells by selective regulation of integrin subunits
Source: J Cell Mol Med. 2018 Dec 3;23(2):1211–23. doi: 10.1111/jcmm.14023 (PMC6349235; doi:10.1111/jcmm.14023)

(A)

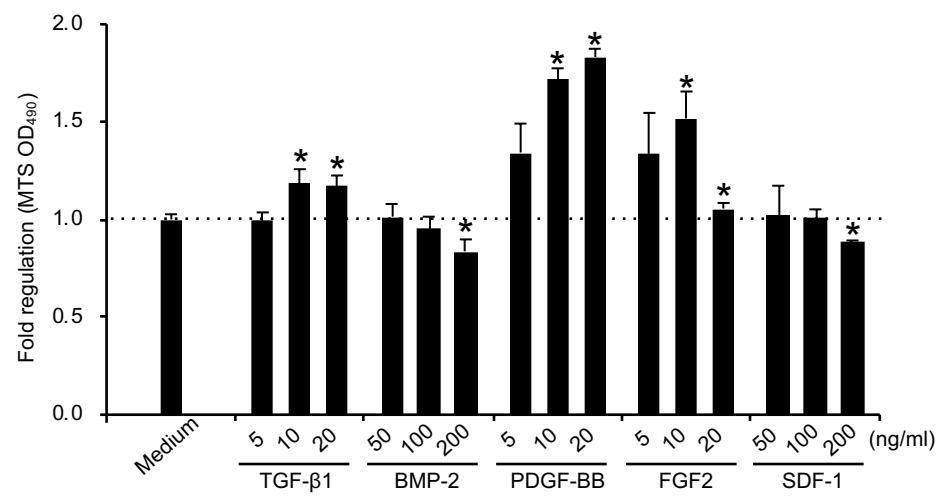

(B)

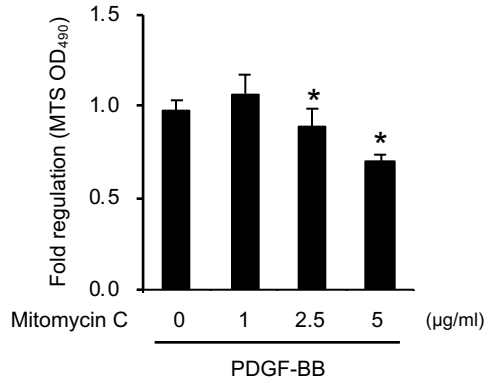

(A)

## PDGF-BB vs. Medium

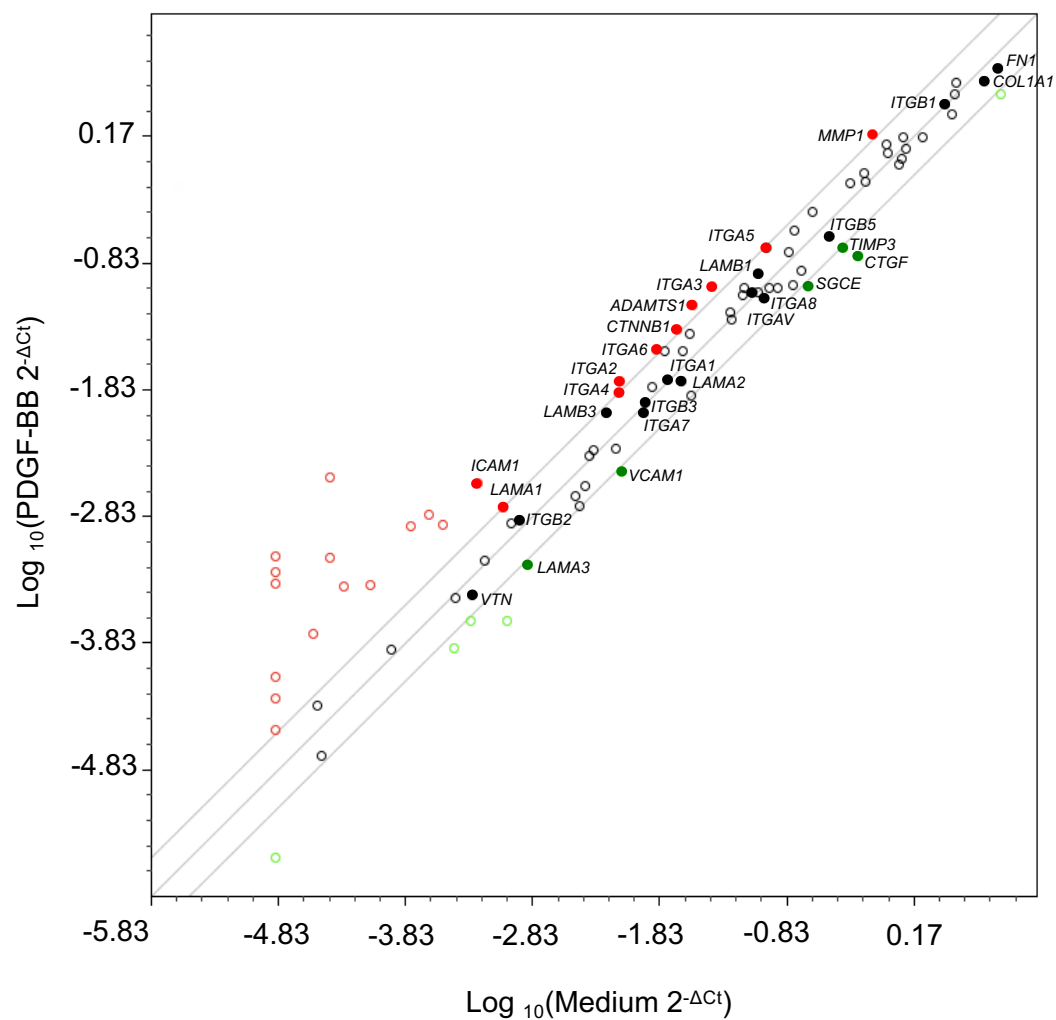

(B)

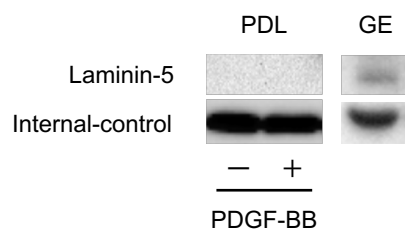

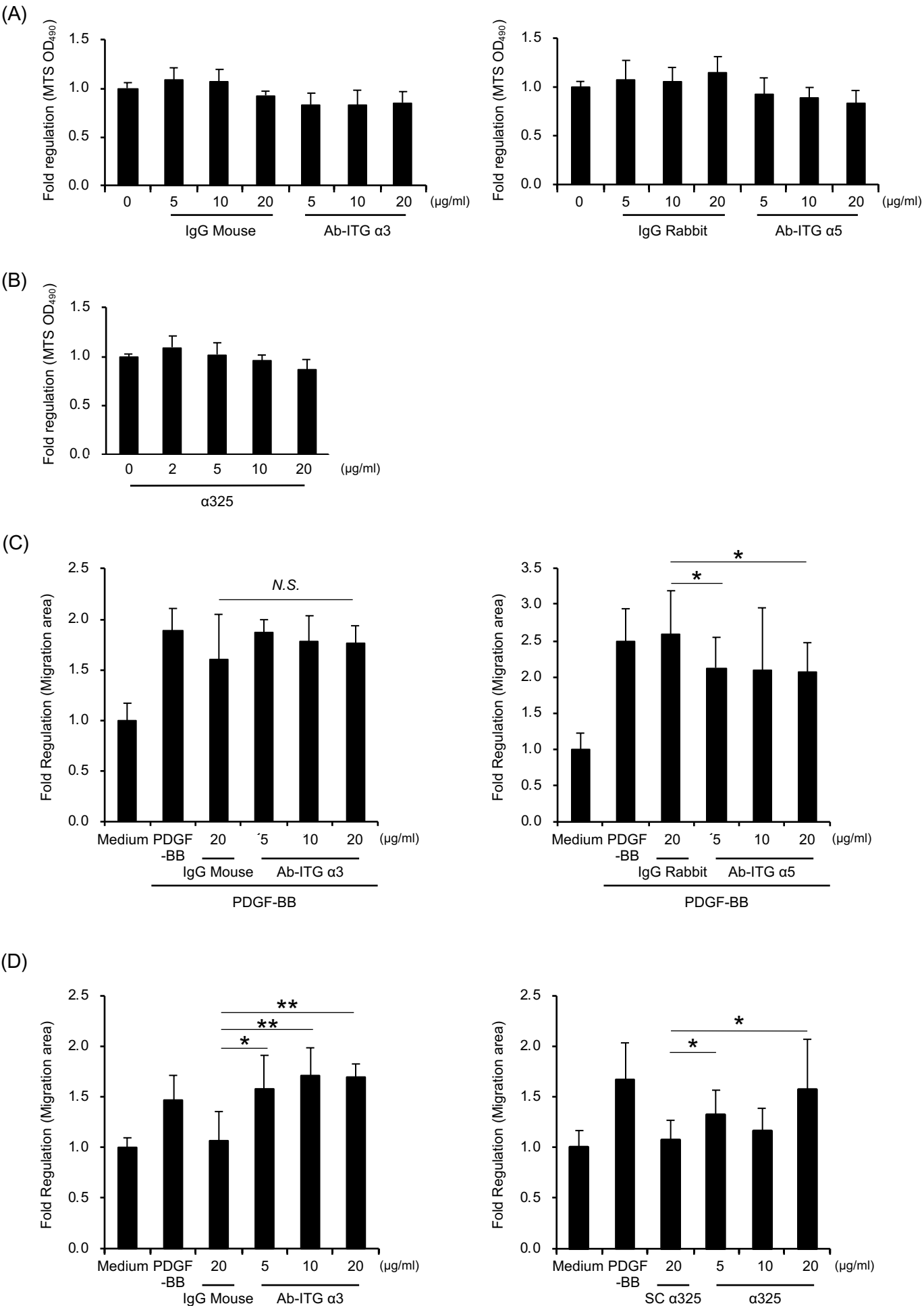

Supplementary Figure 3

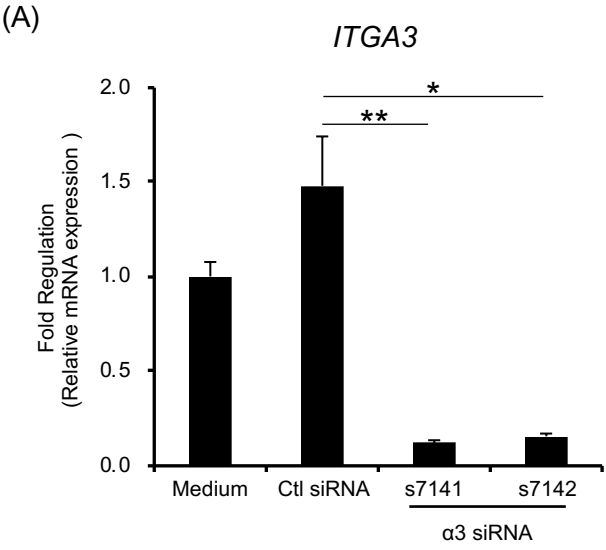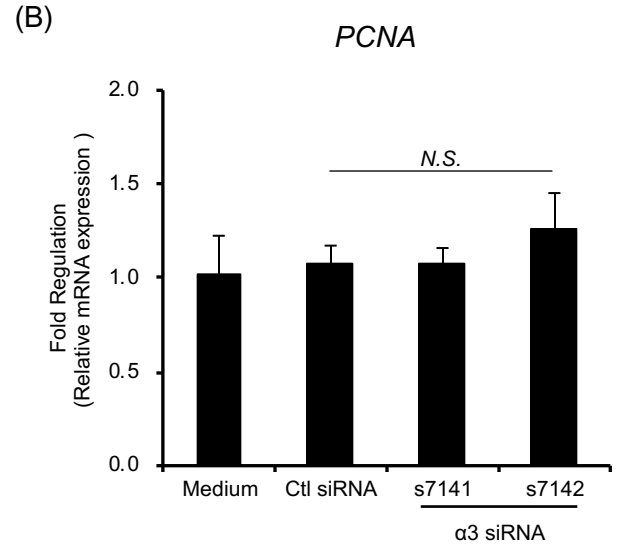

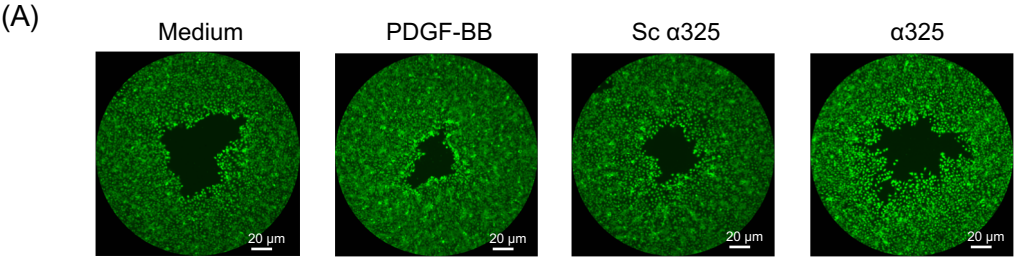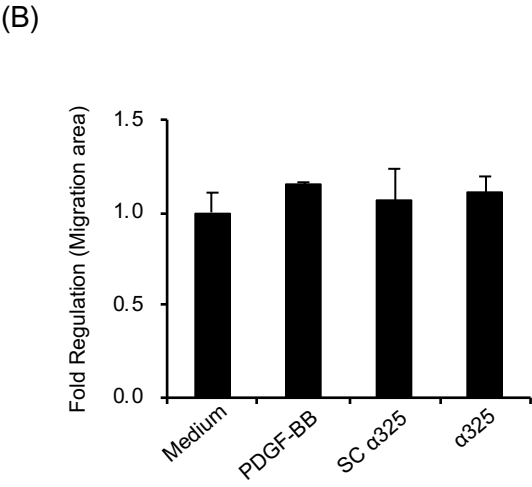

Supplement: Supplementary file 1 [file JCMM-23-1211-s001.pdf]
