# Supplementary material for: Induction of migration of periodontal ligament cells by selective regulation of integrin subunits
Source: J Cell Mol Med. 2018 Dec 3;23(2):1211–23. doi: 10.1111/jcmm.14023 (PMC6349235; doi:10.1111/jcmm.14023)
Supplement: Supplementary file 2 [file JCMM-23-1211-s002.docx]

**Supplementary Table 1 Primer sequences used in this study**

| Gene Symbol | Forward sequence (5'-3') | Reverse sequence (5'-3') |
| --- | --- | --- |
| *ITGA2* | GGGCATTGAAAACACTCGAT | TCGGATCCCAAGATTTTCTG |
| *ITGA3* | GCCTGCCAAGCTAATGAGAC | CACCAGCAGAGTGAGGATCA |
| *ITGA4* | GGGGCGATTTACAGATGCAG | TGGCTGTCTGGAAAGTGTGA |
| *ITGA5* | AGCCTCAGAAGGAGGAGGAC | GGTTAATGGGGTGATTGGTG |
| *ALP* | CCACAGATTTCCCAGCGTCCTTG | GCACCGCCAGCGCCTACC |
| *RUNX2* | CAGACCAGCAGCACTCCATA | GCGTCAACACCATCATTCTG |
| *PCNA* | GAAGCACCAAACCAGGAGAA | TCACTCCGTCTTTTGCACAG |
| *GAPDH* | GAGTCAACGGATTTGGTCGT | GACAAGATTCCCGTTCTCAG |

Glyceraldehyde-3-phosphate dehydrogenase (*GAPDH*) was used as an internal control for real-time RT-PCR analysis in this study. integrin subunit alpha 2 (*ITGA2*), integrin subunit alpha 3 (*ITGA3*), integrin subunit alpha 4 (*ITGA4*), integrin subunit beta 5 (*ITGB5*), alkaline phosphatase (*ALP*), runt-related transcription factor 2 (*RUNX2*), proliferating cell nuclear antigen (*PCNA*).
